# Dopamine and serotonin interplay for valence-based spatial learning

**DOI:** 10.1016/j.celrep.2022.110645

**Published:** 2022-04-13

**Authors:** Carlos Wert-Carvajal, Melissa Reneaux, Tatjana Tchumatchenko, Claudia Clopath

**Affiliations:** 1 Bioengineering Department, Imperial College London, London SW7 2AZ, UK; 2 Theory of Neural Dynamics Group, Max Planck Institute for Brain Research, 60438 Frankfurt, Germany; 3 Institute of Experimental Epileptology and Cognition Research, Life and Brain Center, University of Bonn Medical Center, 53127 Bonn, Germany; 4 Institute of Physiological Chemistry, University of Mainz Medical Center, 55131 Mainz, Germany

**Keywords:** serotonin, dopamine, reinforcement, neuromodulators, hippocampus, navigation, computational, LTD, LTP

## Abstract

Dopamine (DA) and serotonin (5-HT) are important neuromodulators of synaptic plasticity that have been linked to learning from positive or negative outcomes or valence-based learning. In the hippocampus, both affect long-term plasticity but play different roles in encoding uncertainty or predicted reward. DA has been related to positive valence, from reward consumption or avoidance behavior, and 5-HT to aversive encoding. We propose DA produces overall LTP while 5-HT elicits LTD. Here, we compare two reward-modulated spike timing-dependent plasticity (R-STDP) rules to describe the action of these neuromodulators. We examined their role in cognitive performance and flexibility for computational models of the Morris water maze task and reversal learning. Our results show that the interplay of DA and 5-HT improves learning performance and can explain experimental evidence. This study reinforces the importance of neuromodulation in determining the direction of plasticity.

## Highlights

• Plasticity model of DA and 5-HT can reproduce hippocampal valence-based learning
• Interplay of DA and 5-HT has distinct outcomes, whether sequential or additive
• DA and 5-HT learning rules reproduce mouse behavior in the Morris water maze task
• Reversal learning can be represented by antagonism between DA and 5-HT

## Graphical abstract

**Figure:**
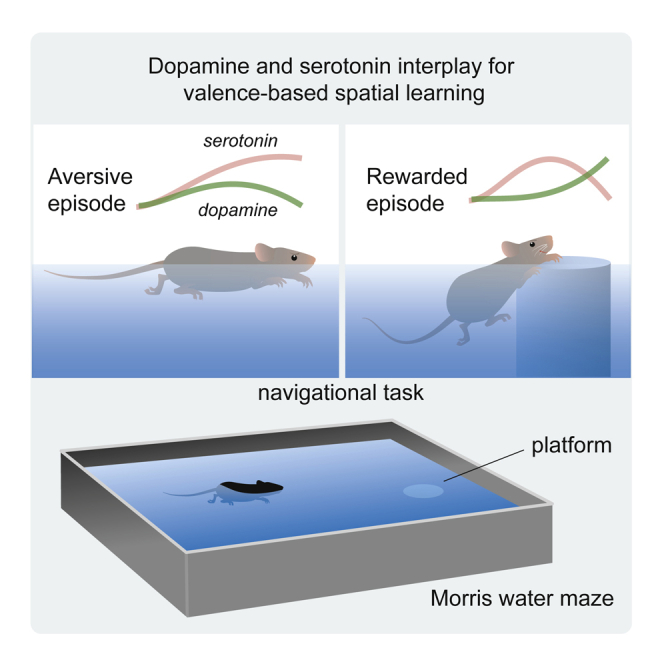

## Teaser

Wert-Carvajal et al. show that a reward-modulated plasticity model of DA and 5-HT antagonism can explain learning by valence in the hippocampus. Their results with optogenetic manipulations of 5-HT reproduce Morris water maze behavior. In addition, they provide experimentally provable predictions regarding reversal learning.

## Introduction

The interplay between dopamine (DA) and serotonin, or 5-hydroxytryptamine (5-HT), regulates cognitive functions underpinning decision making; however, its behavioral consequences and integration into a control system remain elusive (Barnes and Sharp, 1999; Dayan and Huys, 2015). DA has long been characterized as a plasticity modulator that encodes uncertainty or prediction error in reinforcement learning (Schultz et al., 1997; Sutton and Barto, 2018). Unlike other neuromodulators, such as acetylcholine or noradrenaline (Frémaux and Gerstner, 2015), the role of 5-HT is less clear, and it has been hypothesized to contribute to aversive processing, analogous to the function of DA in positive or appetite-driven rewards (Rogers, 2011; Cools et al., 2011; Crockett et al., 2012; Cohen et al., 2015; Fischer and Ullsperger, 2017; Michely et al., 2020). 5-HT also regulates emotional encoding (Rogers, 2011; Dalley and Roiser, 2012), and drugs interfering with 5-HT signaling have been shown to modulate the processing of both rewarding and aversive experiences (McCabe et al., 2010; Fischer and Ullsperger, 2017; Michely et al., 2020). In the hippocampus, a region critical for spatial memory formation (O’Keefe and Dostrovsky, 1971; O’Keefe and Nadel, 1978), DA and 5-HT have been studied in the context of valence-based learning (Fischer and Ullsperger, 2017; Fernandez et al., 2017; Schmidt et al., 2017; Waider et al., 2019). Even if true opponency is not well-established (Daw et al., 2002; Boureau and Dayan, 2011), experimental evidence suggests that the antagonistic effects of DA and 5-HT can explain neural activity during reward-driven learning (Crockett et al., 2012; Cohen et al., 2015; Matias et al., 2017). Notably, DA has been shown to induce long-term potentiation (LTP) in hippocampal goal-directed navigation and avoidance learning (Brzosko et al., 2015; Palacios-Filardo and Mellor, 2019; Broussard et al., 2016). In contrast, 5-HT has been shown to induce long-term depression (LTD) for some receptor-specific hippocampal areas (Kemp and Manahan-Vaughan, 2004, 2005; Berumen et al., 2012; Wawra et al., 2014; Lecouflet et al., 2020). The effect of 5-HT in the hippocampus is still unclear since it has also been observed to produce LTP or metaplasticity regulation (Wang and Arvanov, 1998; Hagena and Manahan-Vaughan, 2017; Teixeira et al., 2018).

Motivated by these experimental findings, we present a mathematical model of valence-based learning in the hippocampus, which details the antagonistic roles of DA and 5-HT for long-term synaptic plasticity. To this end, we use available biological data describing the dynamics of both neuromodulators and present a stable neoHebbian three-factor learning rule (Cassenaer and Laurent, 2012; Frémaux and Gerstner, 2015; Gerstner et al., 2018) characterizing their effect in synapses. By evaluating and optimizing two spike timing-dependent plasticity (STDP) rules during forward learning in a navigational task, we find that 5-HT increases training performance. Finally, we show that the proposed interplay of 5-HT and DA resembles behavioral evidence and can contribute to flexibility during reversal learning (Matias et al., 2017).

## Results and discussion

**Figure 1:**
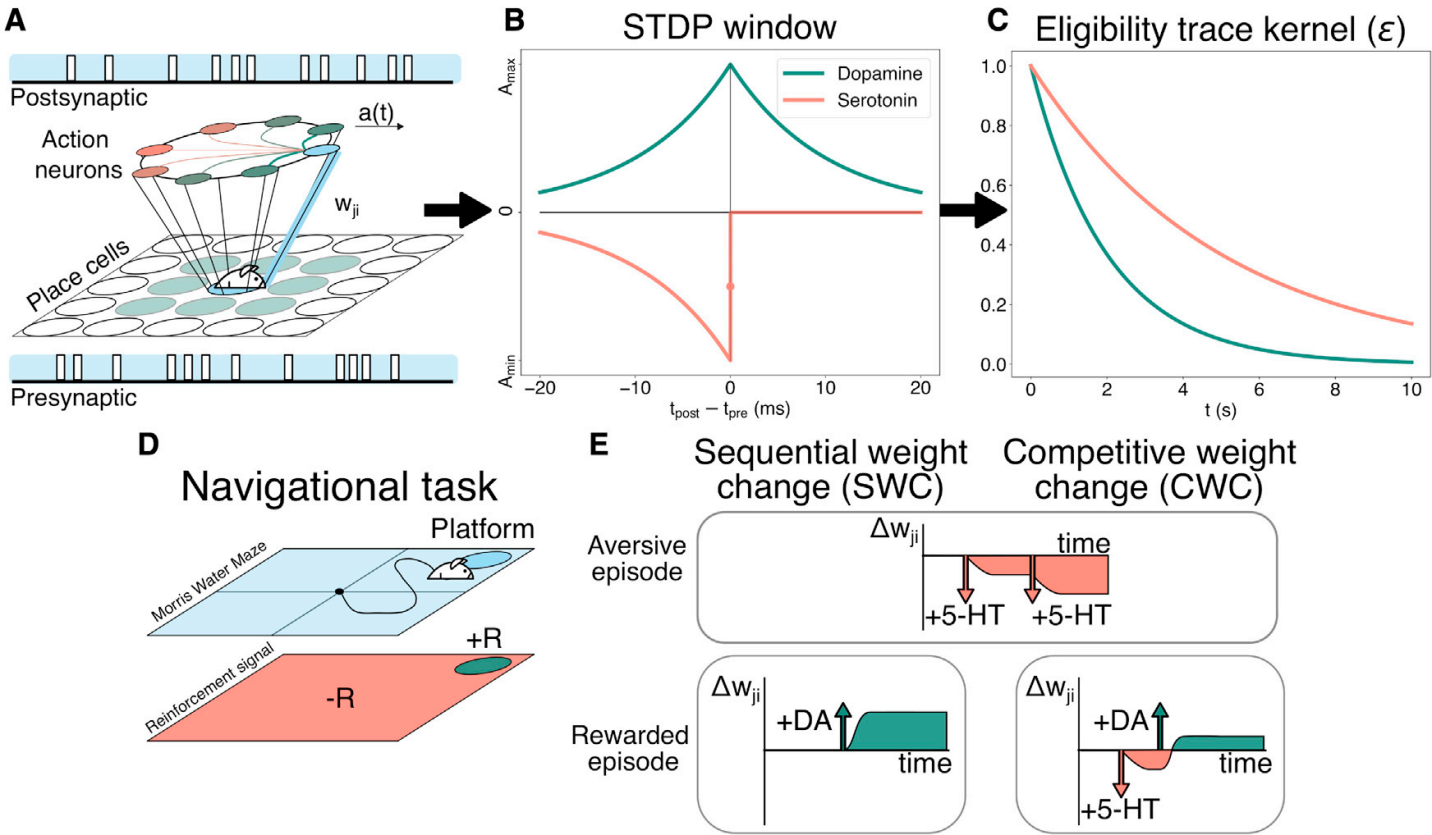
Schematics illustrating CWC and SWC learning rules in our model (A) The navigational model uses a feed-forward network connecting the Gaussian receptive fields to the neurons performing action selection a(t) through “winner-takes-all” connectivity. Forward synaptic weights $w_{ji}$ between neuron pairs (blue) are updated through an R-STDP rule (Equations 8 and 14, STAR Methods). (B) STDP window used for DA and 5-HT. Both neuromodulators have the same STDP time constant; however, the kernel of DA is symmetric and potentiating, while 5-HT presents exclusively anti-causal and depressing contributions. (C) Kernel of an exponentially decaying eligibility trace with experimentally derived time constants. (D) Reward representation derived from water aversion in the MWM task. Negative rewards (-R) are linked to unsuccessful episodes, whereas dopaminergic activation is achieved at platform arrival (+R). (E) Aversive episodes are processed equally by SWC and CWC. Upon reaching a reward, weight updates for SWC are solely driven by DA. Thus, SWC ensures all connections are either potentiated or depressed at the end of an episode. In CWC, rewarded episodes also include aversive cues leading up to the reward. For specific values in (A–C), see STAR Methods.

The valence system we propose for DA and 5-HT contributions highlights the functional importance of the competition between timing-dependent LTP (t-LTP) and depression (t-LTD) during rewarding and aversive reinforcement cues. We followed a navigational hippocampal model (Foster et al., 2000; Vasilaki et al., 2009; Frémaux et al., 2013; Brzosko et al., 2017) with a feed-forward network of presynaptic place cells and a layer of postsynaptic action neurons (Figure 1A; STAR Methods). In reward-modulated spike timing-dependent plasticity (R-STDP), weight changes are based on the spiking timing difference as well as the action of a neuromodulator (Figure 1B). We used previously reported data describing the STDP window for DA in the hippocampus (Brzosko et al., 2015, 2017) and assumed that LTD-inducing effects of 5-HT can be captured by an anti-causal learning window, as shown in cortical 5-HT2C receptors (Figure 1B; He et al., 2015).

Temporal discrimination of neural activity leading up to the reinforcement signal was achieved through an eligibility trace (Gerstner et al., 2018), also known as proto-weight (Huertas et al., 2016) associated with both DA (Brzosko et al., 2017) and 5-HT (Figure 1C; He et al., 2015). Since we adapted the time constant of the eligibility trace of 5-HT from cortical data, we tested alternative decays and outside of a certain temporal range the performance in the Morris water maze (MWM) task is worse (Figure S1D). The neurobiological substrate underpinning eligibility traces has been theorized to be related, for example, to synaptic spine machinery or NMDA receptor-dependent pathways (Gerstner et al., 2018). The latter enables hippocampal DA to retroactively transform STDP into an LTP. This transformation is mediated by receptor subtypes D1 and D2, after a delay associated with memory consolidation of rewarding events (Brzosko et al., 2015). Likewise, hippocampal 5-HT receptors can mediate plasticity by changing calcium levels, as 5-HT4 does by regulating intracellular cyclic AMP and protein kinase A (Barnes and Sharp, 1999; Hagena and Manahan-Vaughan, 2017). The 5-HT2C receptor, on the other hand, influences proteins at the postsynaptic density (Bécamel et al., 2004) to produce LTD conversion (He et al., 2015). Hence, the particular sign of the plasticity change depends on the receptor subtype. Moreover, there is growing evidence of 5-HT intervention in the GABAergic system and transformation of LTP and LTD traces (Lecouflet et al., 2020). The eligibility trace associated with 5-HT, as reported in the neocortex for 5-HT2C, presents a slower decay than with DA in the hippocampus (He et al., 2015). This suggests synaptic “flagging,” or the readiness of a synapse to be potentiated or depressed according to its activity (Gerstner et al., 2018), is less persistent under the modulation of the latter (Figure 1C). In other terms, for an equal synaptic activity, the magnitude of weight change is more attenuated for DA than for 5-HT. Both were studied through standard plasticity pairing protocols and excitatory postsynaptic potential recordings (Brzosko et al., 2015; He et al., 2015). However, observed values of 5-HT in the neocortex are congruent with the degradation of 5-HT in the hippocampus (Saylor et al., 2019).

As a navigational task for our model, we employed an MWM model, where the agent has to find a hidden platform in a water arena (Figure 1D; Vorhees and Williams, 2006). Water is considered an aversive or stress-inducing cue (Harrison et al., 2009), which causes 5-HT release (Karabeg et al., 2013; Sant’Ana et al., 2019). The subsequent arrival at the targeted zone in the corner of the maze produces an increased dopaminergic response (Frémaux et al., 2013), which is crucial for spatial learning (Ploeger et al., 1994; Bernhardt et al., 2018), either by the positive valence associated with the avoidance of a stressor (Antunes et al., 2020; Baik, 2020) or the administration of a reward (Zhai et al., 2007). As a result, we interpreted unrewarded or unsuccessful episodes as those presenting salient aversive or stress-inducing stimuli for this particular setting. Failure to arrive at the platform within a time limit concludes the trial (Vorhees and Williams, 2006). We used the experimentally motivated assumption that more prolonged exposure to water produces a state dominated by negative valence or anxiety (Harrison et al., 2009), whereby 5-HT activation overshadows DA due to the lack of a reward. Hence, in each rule, there is a direct correspondence between behavior and neuromodulatory response, which determines the direction of plasticity (Figure S1A).

We considered two R-STDP learning rules to model DA and 5-HT. First, we implemented sequential weight change (SWC), inspired by sequentially neuromodulated plasticity (sn-Plast) (Brzosko et al., 2017; Zannone et al., 2018), in which DA produces LTP and 5-HT induces LTD during exploration. As sn-Plast, SWC is outcome-dependent and relies on the assumption that potentiation occurs after a delay, which allows decoupling rewarding and non-rewarding episodes (Figure 1E; STAR Methods). This delay could represent the delivery of a caloric reward after the task, also known as consummatory behavior, which increases DA levels (Brzosko et al., 2017). However, there is evidence that, even if the agent does not consume a reward, stress avoidance behavior displays a subsecond DA response upon reaching safety (Oleson et al., 2012). In either case, DA produces the conversion of LTD traces into LTP in the hippocampus (Figure S1A; Brzosko et al., 2015, 2017). In the MWM task, SWC produces t-LTP when the agent finds the platform or t-LTD if it does not, rendering traces as mutually exclusive. It is equivalent to consider that DA dominates over 5-HT if the positive valence item is located, which nullifies the contribution of the stress-inducing cue. Such simplification further relies on the notion that, in addition to being related to stress avoidance, DA also supports goal-directed and error-coding in the hippocampus (Pennartz et al., 2011). Therefore, DA would not be equally present in unsuccessful episodes. Conversely, unrewarded episodes solely present 5-HT modulation, which is performed at the end-of-episode in the case of the MWM. In addition, in opposition to cholinergic depression (Zannone et al., 2018), we adapted SWC to reflect the presence of an eligibility trace for 5-HT. SWC can also present an “online” form of serotonergic modulation with candidate weights being computed continuously, which is equivalent to a step response, without changes in performance (Figure S1C). However, following its sequential nature, the final orientation of the weight change, either LTP or LTD, is resolved by the result of the episode (Figure S1B).

The second learning rule we examined was competitive weight change (CWC), based on competitive reinforcement learning (Figure 1E; Huertas et al., 2016). In contrast to SWC, eligibility traces perform in-episode opposition (Figure S1A), defined mathematically as an addition of DA and 5-HT contributions (see STAR Methods; He et al., 2015), in which the balance between reward-trace pairs determines the weight change. Hence, activated synaptic weights are not guaranteed to be either potentiated or depressed after the reinforcement signal is introduced (Figure S1B). For some configurations, CWC may yield depressive effects upon reaching the platform if DA does not counterbalance 5-HT. Experimental data from serotonergic and dopaminergic neurons suggests differential activity since 5-HT is tonically released as a response to a long-term punishment, and DA has a greater phasic response at the end of a rewarding event (Boureau and Dayan, 2011; Cohen et al., 2015). Hence, we represented transient 5-HT as a step function active until the positive valence item is found, which in turn triggers a DA signal that lasts for less than 1 second according to experimental data (Figure S1A; Cohen et al., 2015; see STAR Methods). The duration and magnitude of DA should be such that it is able to overcome 5-HT-induced LTD while averting weight saturation of non-predictive place-action neuron pairs. For instance, in CWC, the efficiency was lowered when a complete phasic response, modeled as Dirac delta function, was introduced (Figure S1C). In tuning the relative amplitudes of the neuromodulators, we found that the trade-off between the empirically observed phasic response of DA, for which the activation time of DA should be shorter than 5-HT, and DA strength may require a tight adjustment of the DA-5-HT interplay. Consequently, for this navigational task, successful episodes include modulation by both DA and 5-HT, whereas unsuccessful episodes involve exclusively 5-HT. Fundamentally, SWC and CWC diverge in the timing of the weight update, either end-of-episode or in-episode, and in the characterization of DA and 5-HT as alternate or additive (Figures 1E and S1A; see STAR Methods).

**Figure 2:**
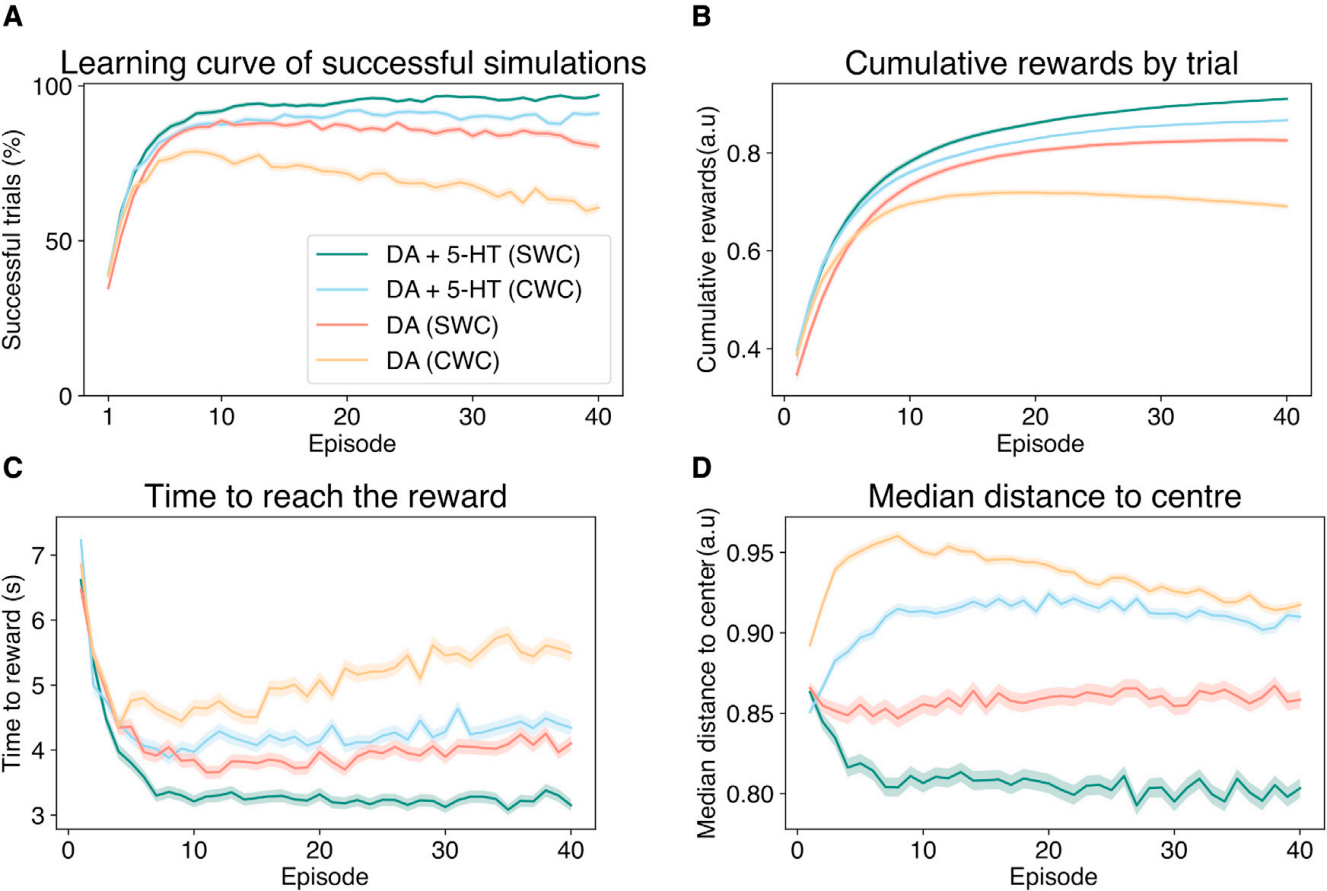
Inclusion of 5-HT improves learning under both R-STDP rules, with enhanced learning for SWC (A) Learning curve for the percentage of successful simulations in each episode. The differences in means between the control (DA) and the addition of 5-HT (DA+5-HT) are significant on the last episode (p<0.01, two-tailed Student’s t test). (B) Cumulative relative number of successful episodes averaged over the simulations. (C) Average latency time to the reward in positive valence episodes. Changes in the time to the reward for the final episode are significant between the different conditions in each rule (p < 0.01, two-tailed Student’s t test). (D) Average median distance to the center as measured in spatial memory tests. The shaded ranges correspond to the standard error of the mean (SEM) in M = 1,000 simulations. See STAR Methods for the value of parameters.

Both models were systematically parametrized through grid search to optimize performance, with final values shown in Table 1. SWC had a better efficiency than CWC in successful simulations over successive trials (Figure 2A) and accumulated successful episodes along trials (Figure 2B). In both cases, the addition of 5-HT as an LTD inducer improved the rewarding outcomes. In CWC, such gain in learning efficiency is sensitive toward the balance between the amplitude of neuromodulators and is preserved for an LTD-only symmetric STDP window, but not if an anti-symmetric LTP contribution is also introduced (Figure S2A), which undoes the enhancement provided by 5-HT by nullifying the area under the curve. In contrast, the equivalent results for SWC reveal this effect is maintained across most magnitudes in strictly depressive functions, which proves this rule is overall less parameter-sensitive (Figure S2B). Hence, our model also proves to be insensitive to spike timing, as it can be inferred by the invariance of performance toward changes in the shape of the STDP window. Moreover, the dependence on the integral of the learning window also holds for an asymmetrical STDP window in DA-only (Figure S2B). As it has been previously suggested (Graupner et al., 2016), the timing independence is due to the high frequency at which spatial information is encoded and the time scales, which makes plasticity dependent solely on the firing rate and the integral of the learning window. As a matter of fact, we can replicate the previous results using a rate-based rule with the same experimental STDP characteristics of DA and 5-HT (Figure S2C; see STAR Methods), which reinforces the importance of the integral instead of precise spike timing statistics. Since the timing of the STDP window of 5-HT does not modify the outcome of simulations (Figure S2D), we chose the time constant of the STDP window of 5-HT to be equal to DA for simplicity.

From a stability perspective, 5-HT restricts the average synaptic weight change across place cells, whereas, if unconstrained by 5-HT, synaptic values converge at increased weight values (Figure S3A). Such convergence corresponds solely to the global stability of the place field. In contrast, individual weights present a higher variability under 5-HT and may not converge to a particular value in the absence of clipping (Figure S3B). Particularly, CWC renders the highest level of overall potentiation in the absence of 5-HT. The saturation of weights can explain the poor performance of DA-only CWC for non-direct paths (Figure S3C), due to the fitting of the reward function and the parameters employed. SWC, in contrast, can yield a greater degree of specificity in weight assignment by potentiating place cells less divergent from the shortest path and depressing synapses yielding non-optimal solutions (Figure S3C). Likewise, the preferential action of each place cell also deviates less from the attraction toward the diagonal, which is the most direct route, than CWC. As a result, compared with SWC, CWC does not optimize the path distance, as measured by the time to reach the reward (Figure 2C) and the median distance to the center with time for both conditions (Figure 2D; Gehring et al., 2015). Varying eligibility trace of 5-HT we found a better shortest path optimization for a more persistent trace in both rules (Figure S1D). However, performance is hindered for time constants deviating from those in the neocortex or of equal magnitude to DA (Figure S1D). Furthermore, performance and path optimization is maintained even if the initial position is randomized between trials (Figure S3D), indicating navigation learning is robust irrespective of the initial location of the agent. To quantify the degree of exploration, we computed the Jensen–Shannon divergence (JSD) between the first reward distribution of both conditions (Zannone et al., 2018; see Quantification and statistical analysis). The divergence in CWC (JSD(+5−HT|−5−HT)=0.075) and SWC (JSD(+5−HT|−5−HT)=0.063) indicates exploration remains unaltered between both cases (Figure S3E). In conclusion, although limited by dynamic and encoding assumptions, we can infer that SWC with 5-HT has a better performance than CWC or any dopamine-only learning rule, and overall provides better path minimization and parameter robustness as compared with CWC.

**Figure 3:**
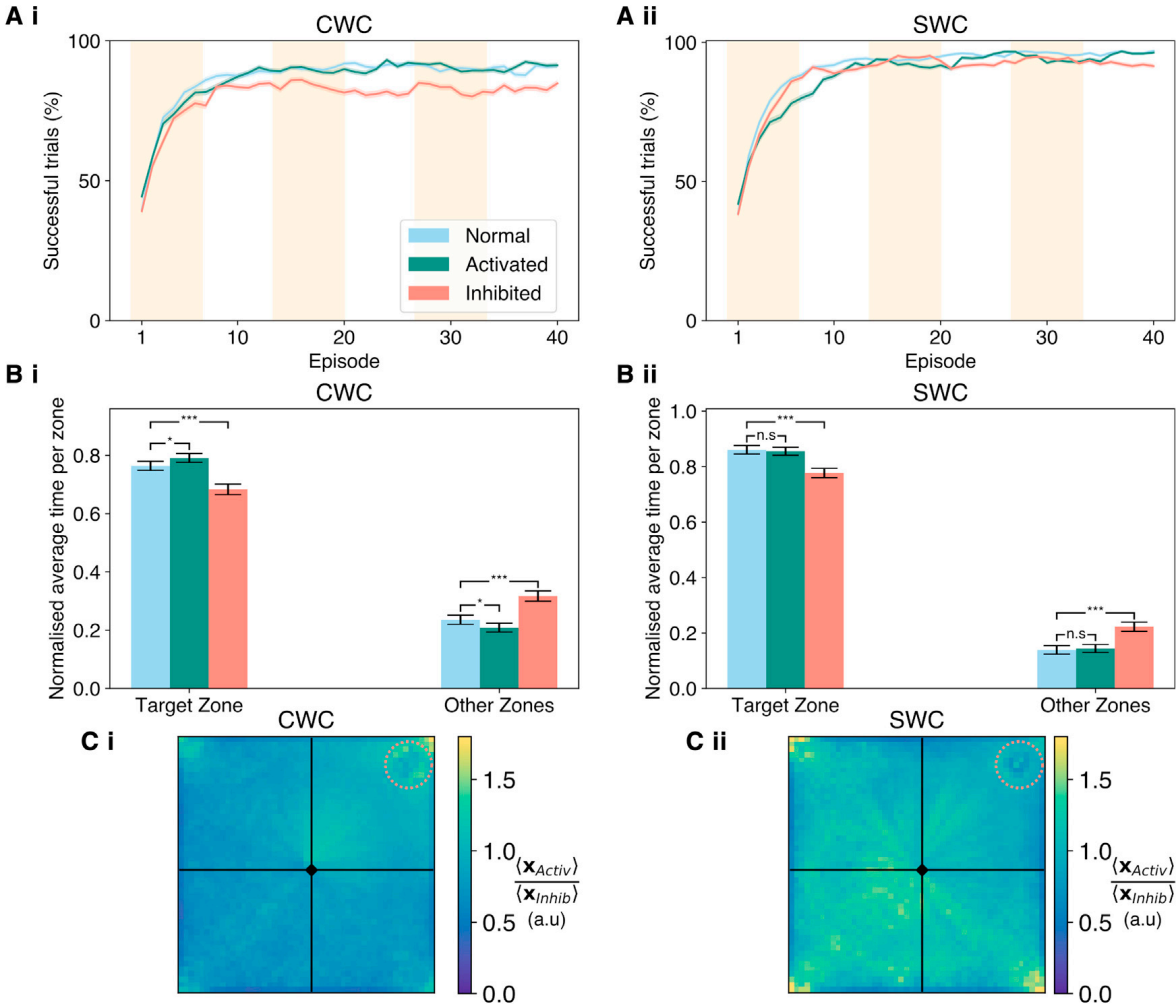
Overactivation and inhibition of 5-HT in CWC are consistent with behavioral data from optogenetic modulation of serotonergic neurons in the MWM (A) Average percentage of successful simulations for i) CWC, and ii) SWC. Episodes in yellow correspond to times in which optogenetic changes are introduced. (B) Bar plot of the average time in the target quadrant against the other zones for i) CWC, which resembles real data observed in mice from Teixeira et al. (2018), and ii) SWC. Statistical significance (two-sample Student’s t test with ^∗^p≤0.05, ^∗∗∗^p≤0.001) is shown for changes in some conditions under paired test. Error bars correspond to a 95% confidence interval. (C) Fold change between activated and inhibited 5-HT averaged over time and simulation position histograms (50 bins per side) for i**)** CWC, and ii) SWC. In an episode, the position is binned spatially then averaged across episodes and trials. The ratio of mean location between conditions is shown with the initial position (rhombus) and the reward location (dotted circle). Filled area and error bars in (A and B) correspond to SEM (M = 1,000).

The biological viability of DA and 5-HT modeling through SWC and CWC was assessed against a study by Teixeira et al. (2018), which demonstrated that optogenetic inhibition and activation of serotonergic neurons modified the learning abilities of mice without significantly affecting locomotion. We aimed to replicate these results by imposing three intervals of the simulation time increasing or omitting the serotonergic signal (Figure 3A), considered as an increase and absence of the punishment, correspondingly. Notably, inhibition of 5-HT in CWC caused a performance reduction, measured as the percentage of successful trials at the final episode. However, overactivation, through a doubling of 5-HT activity, yielded no variation (Figure 3A***.****i*). Similarly, SWC worsened the number of rewarding trials in a restricted configuration with no variation for enhancements of 5-HT levels (Figure 3A***.****ii*). The decline in rewarded outcomes was statistically significant against the control for the last episode in both cases (Figure S4A). These results highlight the importance of 5-HT as a compensatory mechanism of DA in CWC against a more significant role in negative sampling for SWC. For both learning rules, sequential 5-HT inhibition decreased the residence time at the target platform in contrast with the control (Figure 3B). Nevertheless, the increase in serotonergic response only resulted in a larger amount of time spent at the target zone for CWC (Figure 3B *i-ii*). Hence, for time spent in the target quadrant, CWC replicated the changes observed empirically, but SWC did not. In further coherence with their results (Teixeira et al., 2018), we found no significant difference in latency times for CWC, with only a modest deterioration in SWC (Figure S4B). A comparison between the position traces for activation and inhibition shows that 5-HT intensifies movement at the target zone of the maze in CWC (Figure 3C***.****i*), whereas SWC produces a greater divergence in navigation with serotonergic activation enhancing the exploration of non-target quadrants (Figure 3C***.****ii*). Overall, these results suggest that CWC is biological plausible for DA and 5-HT modeling. Nonetheless, additional evidence regarding the activity of serotonergic and dopaminergic neurons during individual simulations could provide a more robust test for the model and corroborate the LTD contribution of 5-HT.

Even if our proposed mechanism could reproduce the behavioral observations by Teixeira et al. (2018), it conflicts with their findings of 5-HT4-mediated LTP in Schaffer collaterals during low delta presynaptic stimulation. In turn, this contrasts with the function assigned to this receptor in CA1 by Lecouflet et al. (2020), in which they report a decrease of LTP magnitude upon 5-HT introduction and theta-burst stimulation, to which our work is closer in frequency terms, as a result of the interplay with the GABAergic system. Interestingly, they also suggest a role of this receptor as an enhancer of LTD magnitude (Lecouflet et al., 2020). This indicates that the action of 5-HT may be dependent on the details of the firing statistics whereby both respond to 5-HT activation but can lead to different plasticity outcomes. Previous work has suggested that this receptor provides differential encoding that depends on regional and frequency-dependent factors (Twarkowski et al., 2016; Hagena and Manahan-Vaughan, 2017). Research by Busceti et al. (2015) and Sant’Ana et al. (2019) concerning 5-HT2C receptors has shown that they are particularly sensitive in the encoding of stressors in mice and that their activity counters or impairs LTP in the hippocampus. These conclusions are relevant for our work since we employed 5-HT2C to infer the STDP window and the eligibility trace dynamics for our model (He et al., 2015). We do not model DA or 5-HT mechanistically; however, we propose that their net downstream effect is antagonist for valence-based learning. Taken together, these studies indicate that 5-HT can result in improved behavioral performance in spatial tasks (Teixeira et al., 2018). Moreover, the particular distributional code underpinning should be actively explored in hippocampal circuits, as is the case of DA (Dabney et al., 2020).

**Figure 4:**
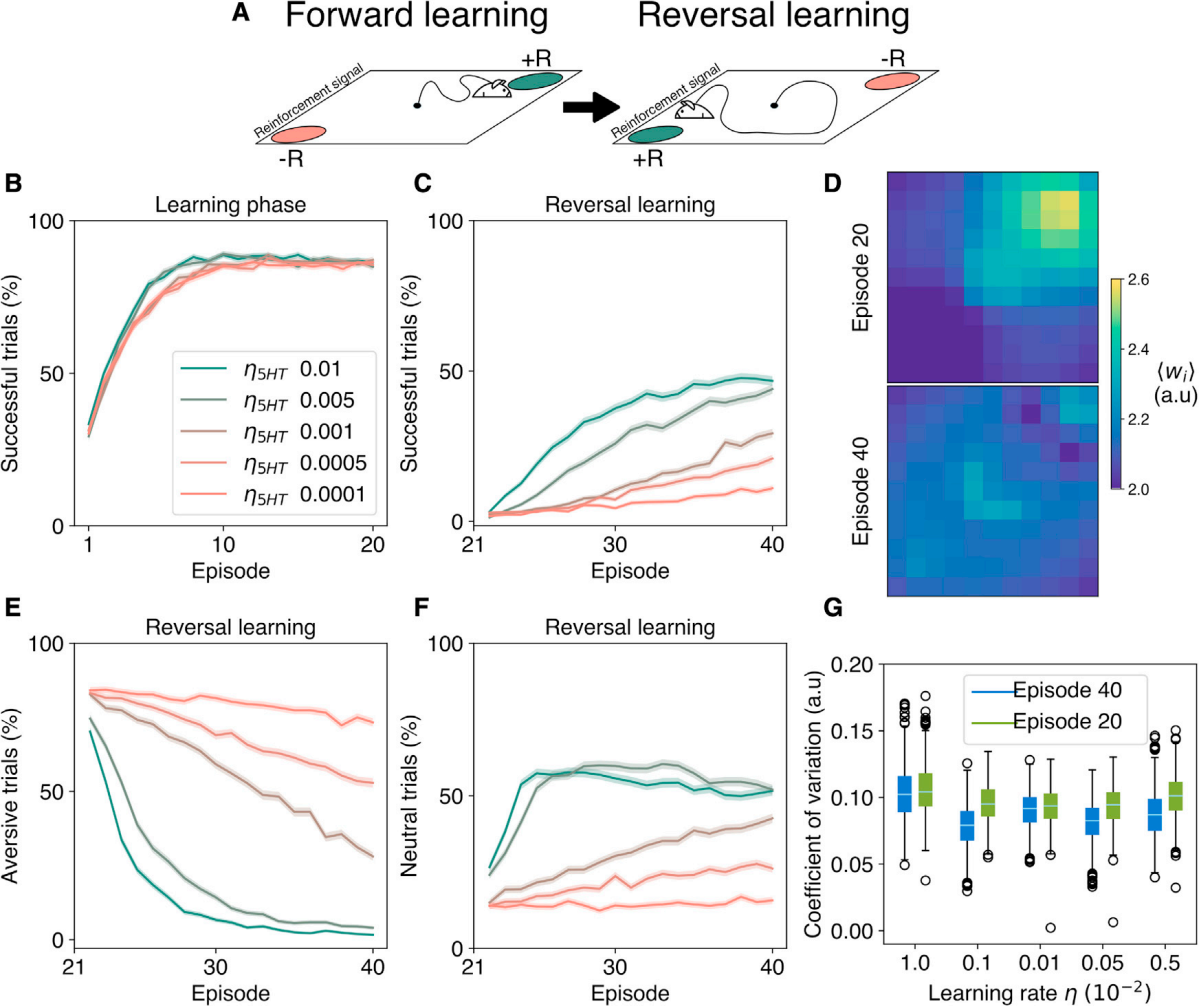
Reversal learning in an open field improves under 5-HT modulation across learning rates (A) Task schematics. The switch between reward and punishment occurs at the middle episode of a trial. (B) Learning curve for the average percentage of successful simulations per trial for five 5-HT learning rates before reversal. (C) Average successful simulations after reversal. (D) Average place cell weights before (episode 20) and after reversal (episode 40) for $\eta_{5HT}=0.01$. (E) Average fraction of punishing or aversive simulations after reversal. (F) Average latency time to reward of successful simulations. (G) Distribution of the mean coefficient of variation (CV) of synaptic weights before and after inversion. This corresponds to the ratio of the standard deviation to the mean of weights in each place cell. Lower CV values after inversion imply a decrease in dispersion or polarization of synaptic weights, as shown in (C). In curves, the shaded region corresponds to SEM (M = 1,000). The error bars in the boxplot are defined with a 95% CI.

The described valence system has been evaluated in reversal learning, which involves punishment and reward switching (Matias et al., 2017). In this setting, t-LTP and t-LTD traces are alternatively present in the weight update for each episode. In other words, since the agent can only reach either a reward or a punishment in an episode, the weight update in CWC has exclusively a positive or a negative valence contribution turning it into a sequential system as in SWC. Hence, we can reconcile SWC and CWC rules into the same system with phasic activity (Figure 4A). In reversal learning, it has been observed that 5-HT operates under a wide range of learning rates, in contrast to DA (Matias et al., 2017). Hence, we evaluated five rates to quantify the effects of an LTD trace in relearning of an environment. For all rates, forward learning was successful (Figure 4B), although there was no recovery after inversion (Figure 4C), with high learning rates performing better. The lack of discrimination is observable in post-reversal synaptic weights compared with forward ones (Figure 4D). In addition, even if negative valence simulations decreased for all learning rates (Figure 4E), most simulations were neutral (Figure 4F), indicating the emergence of a non-decisive or metastable state in deliberation (Bakkour et al., 2019). Reduced selectivity is explained by low polarization in feed-forward weights, as measured by the coefficient of variation (Figure 4G), for all learning rates. Taken together, these results predict a role of 5-HT in aiding reversal learning and present testable conditions in open field navigation under rewarding and punishing cues. However, this model does not explain positive valence encoding of 5-HT observed in conditioning trials (Matias et al., 2017), suggesting a more complex interplay between neuromodulators in hedonic or valence-based learning.

In summary, our plasticity model of interacting DA and 5-HT contributions provides a phenomenological insight into their role in hippocampal-dependent spatial navigation and associates it with valence behaviorally. Moreover, the contrast between SWC and CWC serves as a case of how value assignment depends significantly on the dynamics of the eligibility traces when a neuromodulatory interplay is present. As such, our research suggests that precise temporal discrimination between neuromodulators may improve forward learning performance, as it is shown in SWC. In this sense, the effect obtained through valence-based trial separation or sequential plasticity modulation serves as an ideal case of tight temporal integration between 5-HT and DA in value representation. In contrast, forward learning in CWC indicates that trace superposition can be sub-optimal for performance even if biologically more feasible. These results are of relevance in cases where modulatory antagonism exists, as we theorize for DA and 5-HT in the hippocampus. In spite of the lack of experimental consensus regarding the hippocampal action of 5-HT, our model shows that an opposed regulation of plasticity by its interplay with DA resembles the behavior seen in the MWM. However, our approach could be extended by a clear definition of the response profiles and assumes an independent action by both, motivated exclusively by valence, instead of addressing a possible co-regulation. Moreover, future extensions of this model could explore combinations of aversive and attractive states coded at the level of circuits and a distributional-based approach for the encoding.

### Limitations of the study

Our model assumes simplified and opposite roles for DA and 5-HT in the hippocampus. Such premise is known to be incorrect at the biochemical level, at which both neuromodulators show a functional diversity highly dependent on the receptor type. In particular, the sign of plasticity seems to be influenced by the pathway targeted by DA or 5-HT. Thus, the preponderance of each receptor class may determine the net effect of the interplay in a region and provide a better description of the frequency regimes. However, we interpret our study as a tractable mean-field analysis at the systemic level for its behavioral correspondence.

Furthermore, our approach neglects the role that specific circuitry may play in more instantaneous hedonic credit assignment and decision making. For instance, the ventral hippocampus presents innervations from known centers of emotional response that may be apt for more immediate computations underlying valence-based navigation. In this sense, neuromodulators may act in spatial learning by stabilizing spatial maps, through long-term plasticity, following motivational values.

## STAR★Methods

### Key resources table

**Table:** 

REAGENT or RESOURCE	SOURCE	IDENTIFIER
**Software and algorithms**		

Original code	This paper	https://doi.org/10.5281/zenodo.5841590
Python, version 3.7.9	https://www.python.org	N/A
Numpy, version 1.18.5	https://numpy.org	N/A
Numba, version 0.50.1	https://numba.pydata.org	N/A
Matplotlib, version 3.2.2	https://matplotlib.org	N/A
SciPy, version 1.4.1	https://scipy.org	N/A

### Resource availability

#### Lead contact

Further information and requests should be directed to and will be fulfilled by the lead contact, Claudia Clopath (mailto:cclopath@imperial.ac.uk).

#### Materials availability

This study did not generate new materials.

### Method details

Hippocampal-dependent spatial navigation is modeled through a one-layer network based on a navigational actor-critic system (Frémaux et al., 2013; Brzosko et al., 2017). Location, encoded through the spiking rate of place cells, serves as the input. The output layer is composed of action neurons, which determine the preferred movement of the agent by their firing rate.

#### Place cells

The spiking activity of place cells represents two-dimensional positional information. These are modeled through an inhomogeneous Poisson process with maximum spiking activity ${\bar{\lambda }}^{pc}=400Hz$. The squared norm of the deviation between the Cartesian location of the agent x(t) and the center of the place cell *i* is used for the calculation of the rate as follows:

(Equation 1) $$\lambda_{i}^{pc}\left(x\left(t\right)\right)={\bar{\lambda }}^{pc}\text{exp}\left(-\frac{{\left|\left|x\left(t\right)-x_{i}\right|\right|}^{2}}{\sigma^{2}}\right),$$

The firing rate of the Poisson process exponentially decays with the Euclidean distance between the agent and the center of the place cell. A total of 121 place cells, equally separated by a distance of σ=0.4 a.u., were distributed on a square of length side 4 a.u. (Brzosko et al., 2017).

#### Action neurons

To model action neurons, a zero-order Spike Response Model $\left(SRM_{0}\right)$ was used (Gerstner, 1995), in which the membrane potential is represented as

(Equation 2) $$u_{j}\left(t\right)=\underset{j}{\sum }w_{ji}^{feed}\underset{t_{i}^{f}\in F_{i},t_{i}^{f}>\hat{t_{j}}}{\sum }\epsilon \left(t-t_{i}^{f}\right)+\underset{k,k\neq j}{\sum }w_{jk}^{lat}\underset{t_{k}^{f}\in F_{k},t_{k}^{f}>\hat{t_{j}}}{\sum }\varepsilon \left(t-t_{k}^{f}\right)+\chi \text{Θ}\left(t-\hat{t_{j}}\right)\text{exp}\left(\frac{\hat{t_{j}}-t}{\tau_{m}}\right).$$

In the feed-forward network, the action neuron *j* receives an excitatory postsynaptic potential (EPSP) $\epsilon \left(t-t_{i}^{f}\right)$, from place cell *i*, for firing times in the set $F_{i}$, after the last spike of action neuron $\hat{t_{j}}$ and under a synaptic efficiency $w_{ji}^{feed}$. Similarly, action neurons *k*, of the lateral connectivity network, are connected with synaptic weights $w_{jk}^{lat}$ and their spike arrival times are contained in set $F_{k}$. The EPSP kernel is

(Equation 3) $$\epsilon \left(t\right)=\frac{\epsilon_{0}}{\tau_{m}-\tau_{s}}\left(e^{\frac{-t}{\tau_{m}}}-e^{\frac{-t}{\tau_{s}}}\right)\text{Θ}\left(t\right),$$

where Θ(t) is the Heaviside step function, the membrane constant $\tau_{m}=20$ ms and the rising time $\tau_{s}=5$ ms. (Equation 2) considers a scale factor for the refractory effect χ=−5 mV and (Equation 3) as well with $\epsilon_{0}=20$ mV ms.

The spiking activity is determined by an inhomogeneous Poisson process with rate $\lambda_{j}\left(u_{j}\left(t\right)\right)$, which is formulated as

(Equation 4) $$\lambda_{j}\left(u_{j}\left(t\right)\right)=\lambda_{0}\text{exp}\left(\frac{u_{j}\left(t\right)-\theta }{\text{Δ}u}\right),$$

in which the maximum rate is $\lambda_{0}=60$ Hz, the potential threshold is θ=16 mV and Δu is a voltage window for spike emission that determines the degree of randomness. The dynamics are simplified if the resting potential is assumed to be 0V (Zannone et al., 2018).

The instantaneous firing rate of an action neuron $\rho_{j}\left(t\right)$ is obtained by filtering the spiking activity $Y_{j}=\sum_{t_{j}^{f}\in F_{j}}\delta \left(t-t_{j}\right)$ with the kernel $\gamma \left(t\right)=\frac{e^{\frac{-t}{\tau_{\gamma }}}-e^{\frac{-t}{\nu_{\gamma }}}}{\tau_{\gamma }-\nu_{\gamma }}\text{Θ}\left(t\right)$, where $\tau_{\gamma }=50$ ms and $\nu_{\gamma }=20$ ms.

Each action neuron *k* represents a preferred direction of movement $a_{k}$ and interacts with other angle-encoding action neurons through a lateral connectivity (Frémaux et al., 2013). The lateral synaptic weight dynamics produce a “N-winner-takes-all” arrangement by which $N_{action}$ neurons compete for the preferential angle. Hence, the connectivity between neural units *k* and $k^{\prime }$ is modeled to inhibit opposite directions and excite similar ones as

(Equation 5) $$w_{kk^{\prime }}=\frac{w_{-}}{N_{action}}+w_{+}\frac{f\left(k,k^{\prime }\right)}{\sum_{k^{\prime }}f\left(k,k^{\prime }\right)},$$

inhibitory and excitatory weights are $w_{-}=-300$ and $w_{+}=100$ and *f* is the lateral connectivity function, which reaches a maximum for $k=k^{\prime }\pm 1$ and decreases monotonically towards zero for $k=k^{\prime }$. Concretely, in this case, $f\left(k,k^{\prime }\right)=\left(1-\delta_{k,k^{\prime }}\right)\text{exp}\left(\zeta \text{cos}\left(\theta_{k}-\theta_{k^{\prime }}\right)\right)$ decreases exponentially for increasingly dissimilar angles $\theta_{k^{\prime }}$ and is scaled by a factor ζ=20. These parameters were tuned for a population of $N_{action}=40$ with $\theta_{k}=\frac{2k\pi }{N_{actor}}$ (Zannone et al., 2018). Thus, action vectors were $a_{k}=a_{0}{\left(\text{sin}\left(\theta_{k}\right),\text{cos}\left(\theta_{k}\right)\right)}^{T}$ with $a_{0}=0.08$.

The action resulting from the spiking activity of the network is coded through a population vector as

(Equation 6) $$a\left(t\right)=\frac{1}{N_{action}}\underset{k}{\sum }\rho_{k}\left(t\right)a_{k},$$

which is weighted by the filtered spiking activity of neurons $\rho_{k}\left(t\right)=\left(Y_{k}○\gamma \right)\left(t\right)$. Thus, the action at each time step a(t) is computed as the average of the action vectors with the predicted instantaneous activity of actor neurons. The inertia of movement is determined by the activity of the network with the maximum velocity being limited by $a_{0}$.

#### Navigational setting

The square *S* delimiting the two-dimensional plane, serves as a boundary condition for the position x(t) and the movement of the agent a(t). This is formulated as

(Equation 7) $$\Delta x\left(t\right)=\left\{ \begin{array}{ll} a\left(t\right) & \forall x\left(t+1\right)\in S|S=\left\{\left[-2,2\right]\times \left[-2,2\right]\right\}\\ d\left(t\right) & \text{else}. \end{array}\right.$$

The bouncing vector d(t) corresponds to the displacement of the agent in the direction of the normal vector to *S* or $n_{S}$, which is defined as $d\left(t\right)=d_{0}n_{S}\left(x\left(t\right)\right)$. The bouncing distance is set as $d_{0}=0.01$.

In the MWM, the rewarding platform was positioned at $r_{c}=\left(1.5,1.5\right)$ with a radius $r_{r}=0.3$. In reversal learning, the reward is initially maintained at its position and the punishment is placed at $p_{c}=\left(-1.5,-1.5\right)$ with radius $p_{r}=0.3$, for episodes 1 to 20. After reversal, both elements switch position, $r_{c}=\left(-1.5,-1.5\right)$ and $p_{c}=\left(1.5,1.5\right)$, with unvaried radii, for episodes 21 to 40. In all instances, the initial position of the agent was x(t=0)=(0,0) and the maximum episode time was $T_{max}=15$ s (Zannone et al., 2018). If the goal is reached before $T_{max}$, the episode is ended, and place cells are deactivated. In SWC, the weight update occurs at $t=T_{rew}+300$ ms, to replicate consummatory behavior. Per contra, in CWC, weights are updated continuously until the DA signal is no longer active $t=T_{rew}+T_{DA}$. Activity is reset between episodes.

#### Sequential weight change (SWC)

The sequentially neuromodulated plasticity (sn-Plast) rule from Brzosko et al. (2017) was adapted to match the empirical evidence available for 5-HT in He et al. (2015). Hence, instead of presenting an online depression mediated by acetylcholine, the adjusted SWC update introduces an eligibility trace $\varepsilon_{5HT}$ for 5-HT which considers the neural activity leading up to an aversive cue. As for DA, we maintained the characteristics of the trace (Brzosko et al., 2017). Accordingly, the eligibility traces of DA and 5-HT are formulated as

(Equation 8) $$\epsilon \left(t\right)=e^{\frac{-t}{\tau_{e}}}\text{Θ}\left(t\right).$$

We used the previously reported value of the time constant for DA (Brzosko et al., 2017) while for 5-HT we employed values observed *in vivo* in the neocortex (He et al., 2015). In particular, these are $\tau_{e}=\left\{ \begin{array}{ll} 2\text{ s} & \text{for DA}\\ 5\text{ s} & \text{for }5-\text{HT} \end{array}\right.$.

The disparity between these time constants introduces a differential response for an equal predictive neural activity. In other terms, place cells encoding for the same path suffer a greater weight change, in absolute terms, through serotonergic action than with DA modulation for an equal STDP response.

Following Brzosko et al. (2017), we used a symmetric STDP window for DA, defined as

(Equation 9) $$W_{DA}\left(s\right)=A_{DA}e^{\frac{-|s|}{\tau_{DA}}},$$

where $\tau_{DA}=10$ ms and $A_{DA}$ is the amplitude of the window. For 5-HT the STDP window is strictly depressive and asymmetric (He et al., 2015) and takes the form

(Equation 10) $$W_{5HT}\left(s\right)=\left\{ \begin{array}{ll} A_{5HT}e^{\frac{-s}{\tau_{5HT}}} & \text{if }s\;>\;0\\ \frac{1}{2}A_{5HT} & \text{if }s=0\\ 0 & \text{if }s\;<\;0, \end{array}\right.$$

in which $A_{5HT}$ is the magnitude of the window for 5-HT. For simplicity, 5-HT induces LTD through an auxiliary variable *R* that switches the sign of the update. Hence, for sign convention, we use $A_{5HT}\;>\;0$, but the observed STDP window of 5-HT is negative.

The weight update is determined by the STDP window of each neuromodulator W(s) filtered by its eligibility trace ε, and an outcome-dependent signal *R*. Taken together, the weight change of our model is

(Equation 11) $$\Delta w_{ji}\left(t\right)=\eta R\left(\left(\underset{t_{i}^{f}\in F_{i}^{pc}}{\sum }\underset{t_{j}^{f}\in F_{j}^{a}}{\sum }W\left(t_{j}^{f}-t_{i}^{f}\right)\right)○\epsilon \right)\left(t\right),$$

with the firing times $t_{j}^{f}$ and $t_{i}^{f}$ of action neuron *j* and place cell *i* contained in the sets $F_{j}^{a}$ and $F_{i}^{pc}$. The learning rate η is different for 5-HT and DA, such that $\eta =\left\{ \begin{array}{ll} \eta_{DA} & +\text{DA}\\ \eta_{5HT} & +5-\text{HT} \end{array}\right.$. Only feed-forward weights are modified by this rule, since synapses between action neurons are updated according to (Equation 5).

By construction, *R* also encodes the overall valence of the simulation and, thus, the action of the dominating modulator. Hence, $R=\left\{ \begin{array}{ll} 1 & +\text{DA}\\ -1 & +5-\text{HT} \end{array}\right.$.

Our SWC model assumes that, at the end of each episode, either DA or 5-HT has been predominant in the simulation and, thus, only one is considered for computing the synaptic weight change. Specifically, in the MWM, DA is released if the agent reaches the reward. At the same time, in an unrewarded episode, aversiveness towards water causes 5-HT to be more salient in those where the platform is not reached. This representation holds for the reversal learning task, where the outcomes (i.e., reward and punishment) are mutually exclusive, that is, either DA or 5-HT is active. To produce stable maps, we clipped synaptic weights between $w_{min}$ and $w_{max}$ at the end of each episode. The values are shown in Table 1.

**Table 1:** Parameters used for SWC and CWC models

Model	$A_{DA}$	$A_{5HT}$	η	$w_{min}$	$w_{max}$	$R_{DA-Amp}$	$R_{5HT-Amp}$	$\tau_{STDP}$	$\tau_{\varepsilon }\left(DA\right)$	$\tau_{\varepsilon }\left(5HT\right)$
CWC	1	0.01	0.0001	1	3	1	1	10 ms	2 s	5 s
SWC	1	1	0.01	1	3	–	–	10 ms	2 s	5 s

These parameters correspond to the best models with regard to the percentage of successful simulations in the last trial.

For serotonergic depression the weight update can be computed either at the end of the episode or continuously along the trial, without modifying the performance, as long as the weight assignment is determined by the outcome of the episode. For the continuous case, we decreased the learning rate by $\eta_{5HT-online}=\frac{1}{T_{max}}\eta_{5HT}$.

For the plasticity rule using firing rates, we adhered to a BCM implementation that follows directly the previous STDP characteristic (Izhikevich and Desai, 2003). We used strictly Hebbian and anti-Hebbian contributions for DA and 5-HT, respectively, which can be formulated as

(Equation 12) $$W\left(\nu_{post},\nu_{pre}\right)=\left(\frac{A_{STDP}}{\tau^{-1}+\nu_{post}}\nu_{post}\nu_{pre}\right)\Rightarrow \dot{w_{ji}}\left(t\right)=\eta R\left(\left(\frac{A_{STDP}}{\tau^{-1}+\nu_{j}}\nu_{j}\nu_{i}\right)○\epsilon \right),$$

where $A_{STDP}$ and τ are the amplitude and decay of the STDP window for each neuromodulator, as indicated previously (Equations 9 and 10), $\nu_{j}$ is the rate of an action neuron *j*, as obtained from (Equation 3), and $\nu_{i}$ is the frequency of a place cell *i* as calculated in (Equation 1). As in its STDP form, *R* encodes the outcome or general valence of the simulation and, hence, produces a switch between the Hebbian and anti-Hebbian cases of the rule.

#### Competitive weight change (CWC)

In competitive weight change (CWC), inspired by competitive reinforcement learning from Huertas et al. (2016), the eligibility traces of DA and 5-HT are engaged in a dynamic competition for the upgrade of the synaptic weight.

Firstly, the STDP windows W(s) of DA (Equation 9) and 5-HT (Equation 10) are convolved with their respective eligibility trace kernels ε (Equation 8), for each pre-post pair of neurons *j* and *i*, leading to a proto-weight Γ described as

(Equation 13) $$\begin{array}{l} \text{Γ}\left(t\right)=\left(W\left(s\right)○\epsilon \right)\left(t\right),\\ {\text{Γ}}_{ji}\left(t\right)=\left(\left(\underset{t_{i}^{f}\in F_{i}^{pc}}{\sum }\underset{t_{j}^{f}\in F_{j}^{a}}{\sum }W\left(t_{j}^{f}-t_{i}^{f}\right)\right)○\epsilon \right)\left(t\right). \end{array}$$

The weight update $\dot{w_{ji}}\left(t\right)$ becomes a dynamical subtraction of t-LTP and t-LTD proto-weights

(Equation 14) $$\dot{w_{ji}}\left(t\right)=\eta \left(R_{DA}\left(t\right){\text{Γ}}_{DA}-R_{5HT}\left(t\right){\text{Γ}}_{5HT}\right),$$

where ${\text{Γ}}_{DA}$ and ${\text{Γ}}_{5HT}$ are the proto-weights of t-LTP and t-LTD, respectively. $R_{DA}\left(t\right)$ and $R_{5HT}\left(t\right)$ model the response or activation function of the neuromodulators subject to a learning rare η.

The integral version of (Equation 14) is

(Equation 15) $$\Delta w_{ji}\left(t\right)=\eta \left(R_{DA}\left(t\right)\left(\left(\underset{t_{i}^{f}\in F_{i}^{pc}}{\sum }\underset{t_{j}^{f}\in F_{j}^{a}}{\sum }W_{DA}\left(t_{j}^{f}-t_{i}^{f}\right)\right)○\epsilon_{DA}\right)\left(t\right)-R_{5HT}\left(t\right)\left(\left(\underset{t_{i}^{f}\in F_{i}^{pc}}{\sum }\underset{t_{j}^{f}\in F_{j}^{a}}{\sum }W_{5HT}\left(t_{j}^{f}-t_{i}^{f}\right)\right)○\epsilon_{5HT}\right)\left(t\right)\right),$$

where the reinforcement signals are modeled as Heaviside step functions Θ(t). Hereby, $R_{DA}\left(t\right)=R_{DA-Amp}\left(\text{Θ}\left(t-T_{rew}\right)-\text{Θ}\left(t-T_{DA}-T_{rew}\right)\right)$ and $R_{5HT}\left(t\right)=R_{5TH-Amp}\left(\text{Θ}\left(t\right)-\text{Θ}\left(t-T_{rew}\right)\right)$. Accordingly, 5-HT is active until the reward is obtained or, in the MWM, the trial has ended unsuccessfully, we denote both times as $T_{rew}$. To mimic a more time-limited response of DA (Cohen et al., 2015) and consummatory behavior (Brzosko et al., 2017), DA acts for a time after the arrival of the agent to the positive reinforcement site, which is $T_{DA}=1$ s. The ratio between the R_DA-Amp_ and R_5HT__-Amp_ is expressed as R_D._

$$R_{D}=\frac{R_{DA-Amp}}{R_{5HT-Amp}}$$

In contrast to Huertas et al. (2016), at the end of the simulation, the potentiation or depression of flagged synapses (i.e., neural connections that have been active during the trial) is not necessarily consistent with the outcome of the trial. Hence, the condition to obtain LTP or LTD becomes

(Equation 16) $$\begin{array}{l} R_{DA}\left(t\right){\text{Γ}}_{DA}\;>\;R_{5HT}\left(t\right){\text{Γ}}_{5HT}\Rightarrow \dot{w_{ji}}\left(t\right)\;>\;0\;\left(LTP\right)\\ R_{DA}\left(t\right){\text{Γ}}_{DA}\;<\;R_{5HT}\left(t\right){\text{Γ}}_{5HT}\Rightarrow \dot{w_{ji}}\left(t\right)\;<\;0\;\left(LTD\right) \end{array}$$

Accordingly, for example, at the end of a successful episode the net synaptic change could be depressive for some connections. Notably, in our MWM implementation, the condition for weight potentiation after reaching the platform requires that

(Equation 17) $$\Delta w_{ji}\left(t=T_{episode}\right)\;>\;0\Leftrightarrow \int_{0}^{T_{episode}}R_{DA}\left(t\right){\text{Γ}}_{DA}\left(t\right)\;>\int_{0}^{T_{episode}}R_{5HT}\left(t\right){\text{Γ}}_{5HT}\left(t\right)\;,$$

In which Γ is the proto-weight of the respective neuromodulator and *R* the reinforcement signal. It should be noted that this formulation depends on the time of the episode $T_{episode}$ and, therefore, the sign of weight update values can vary between trials of different duration. This is equivalent, in integral terms, to Equation 16. As in SWC, we clipped weights according to values in Table 1.

As shown by Huertas et al. (2016), the formulation in (Equation 13) can be used for reward or cue timing learning if the potentiating and depressive responses coincide in time and the decay of the eligibility trace of LTD is faster than LTP, which we did not assumed. This means that a network may learn the underlying dynamics of a cue or signal through the dynamic competition of its traces. However, due to the constraints and assumptions of our system, we only modeled in-episode competition for the final weight update through the superposition of LTP and LTD proposed in their analysis (Huertas et al., 2016).

#### Parameter values

The configuration of each model was optimized through grid search parametrization. In particular, we optimized the amplitudes of the STDP widows, $A_{DA}$ and $A_{5HT}$, the reward magnitudes for CWC $R_{DA-Amp}$ and $R_{5HT-Amp}$, and the learning rates η. Sweeps of these values were first conducted by orders of magnitude and then with fine tuning around good estimates. The best models were selected by the proportion of successful simulations at the final trial.

Similarly to SWC, the rate-based approach of CWC adapted the STDP window to follow the frequencies of the presynaptic and postsynaptic components. Hence, the proto-weights of (Equation 13) show a dependence on the Hebbian and anti-Hebbian formulations in (Equation 12), for DA and 5-HT respectively.

### Quantification and statistical analysis

#### Quantification

All simulations were performed in a specified number of parallel simulations (M) each presenting a number of consecutive episodes.

#### Statistical analysis

All statistical tests are reported with the corresponding significance level and the number of trials. Metrics dependent on the simulation outcome (e.g., time to reward), were adjusted for a variable sample size. Significance levels in plots correspond to p≤0.05, ^∗^; p≤0.01, ^∗∗^; p≤0.001, ^∗∗∗^; p≥0.05, n.s.

The Jensen-Shannon divergence (JSD) between two distributions *P* and *Q* is computed as

(Equation 18) $$JSD\left(P||Q\right)=\frac{1}{2}D_{KL}\left(P||M\right)+\frac{1}{2}D_{KL}\left(Q||M\right),$$

where $M=\frac{1}{2}\left(P+Q\right)$. The metric is symmetric (i.e., JSD(P||Q)=JSD(Q||P)) and is bounded between 0 and 1, which correspond respectively to identical distributions or maximally different. $D_{KL}$ is the Kullback–Leibler divergence as obtained from

(Equation 19) $$D_{KL}\left(P||Q\right)=\underset{x\in X}{\sum }P\left(x\right){\text{log}}_{2}\left(\frac{P\left(x\right)}{Q\left(x\right)}\right).$$

The coefficient of variation (CV) is a normalised measure of variability or dispersion. The population CV is characterized as $\hat{CV}=\frac{s}{\bar{x}}$, where $\bar{x}$ and *s* are the sample average and standard deviation, respectively. For each simulation, the CV is obtained from the distribution of weights at a particular episode, which is then averaged across trials.

Distance statistics where estimated from a metric in each episode, such as the median distance to the center, or resulted from quantization to discrete values in order to reduce storage and produce comparable results between conditions.

#### Software

All software used (Python, Matplotlib, Numba, Numpy and SciPy) is freely available. The specific versions used are listed in key resources table.

## Data Availability

• Datasets to produce the main figures are publicly available at https://doi.org/10.5281/zenodo.5841590. • All original code has been deposited at https://doi.org/10.5281/zenodo.5841590 and is publicly available as of the date of publication. • Any additional information required to reanalyze the data reported in this paper is available from the lead contact upon request.

## Supplemental information

Document S1. Figures S1–S4

Document S2. Article plus supplemental information
