# Supplementary material for: Dopamine and serotonin interplay for valence-based spatial learning
Source: Cell Rep. 2022 Apr 13;39(2):110645. doi: 10.1016/j.celrep.2022.110645 (PMC9620746; doi:10.1016/j.celrep.2022.110645)
Supplement: Document S1. Figures S1–S4 [file mmc1.pdf]

Cell Reports, Volume *39*

Supplemental information

Dopamine and serotonin interplay

for valence-based spatial learning

Carlos Wert-Carvajal, Melissa Reneaux, Tatjana Tchumatchenko, and Claudia Clopath

Supplemental Information

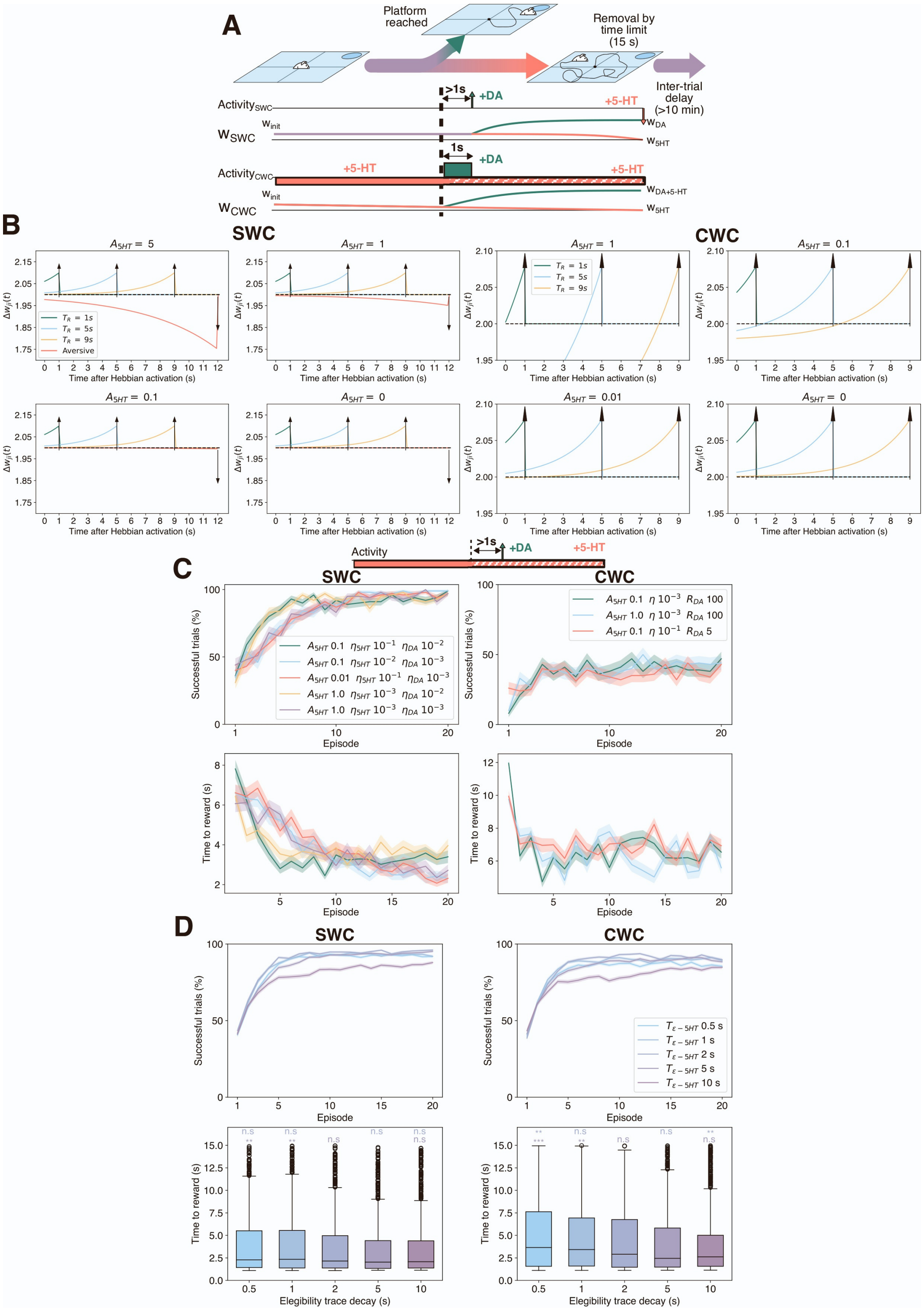
A
Platform reached
Removal by time limit (15 s)
Inter-trial delay (>10 min)
Activity_SWC
>1s
+DA
+5-HT
w_init
W_SWC
w_DA
w_5HT
Activity_CWC
1s
+5-HT
+DA
+5-HT
w_DA+5-HT
W_CWC
B
SWC
CWC
A_5HT = 5
A_5HT = 1
A_5HT = 0.1
A_5HT = 0
A_5HT = 0.01
T_R = 1s
T_R = 5s
T_R = 9s
Aversive
Δw_ji(t)
Time after Hebbian activation (s)
C
Activity
>1s
+DA
+5-HT
SWC
CWC
A_5HT 0.1 η_5HT 10^-1 η_DA 10^-2
A_5HT 0.1 η_5HT 10^-2 η_DA 10^-3
A_5HT 0.01 η_5HT 10^-1 η_DA 10^-3
A_5HT 1.0 η_5HT 10^-3 η_DA 10^-2
A_5HT 1.0 η_5HT 10^-3 η_DA 10^-3
A_5HT 0.1 η 10^-3 R_DA 100
A_5HT 1.0 η 10^-3 R_DA 100
A_5HT 0.1 η 10^-1 R_DA 5
Successful trials (%)
Time to reward (s)
Episode
D
SWC
CWC
T_ε-5HT 0.5 s
T_ε-5HT 1 s
T_ε-5HT 2 s
T_ε-5HT 5 s
T_ε-5HT 10 s
Successful trials (%)
Episode
Time to reward (s)
Elegibility trace decay (s)
n.s
**
***

Figure S1: **Evolution of the plasticity rules in the MWM task and temporal details. Related to Figure 1.**. **A** Activity traces and synaptic weights according to SWC and CWC. In the MWM, the agent must find a hidden platform (dashed line), within a specific time limit, or it is removed from the field. In a successful episode DA is released whereas task failure involves solely 5-HT activity. We use inter-trial time higher than 10 min to assume an activity separation along episodes Brzosko et al. (2015). In SWC, the activity trace corresponds to either 5-HT, at the end of the episode, or phasic DA, delivered with a delay to model consummatory behavior. For CWC, 5-HT is assumed to be constant until the platform is discovered (patterned box), when, in turn, DA becomes active with a time-limited step response. **B** Weight change as a function of time for SWC and CWC for three rewarding times (arrows) and an aversive stimulus. Instead of using spike timing, the Hebbian term is fixed as a Dirac delta function $W(t) = \delta(t)$. Since 5-HT is only active if the time limit is reached, SWC updates are sign-consistent which CWC does not guarantee as the aversion is constant in the MWM. **C** Performance as percentage of successful trials over successive simulations and latency time to the platform for a equal activity functions of DA and 5-HT (above). SWC is able to preserve performance under a normalized constant step function as 5-HT but CWC has a hindered efficiency with a Dirac delta DA response. Combinations correspond to the best out of nine tested (M=100 simulations). **D** Performance in CWC and SWC for different time constants of the proto-weights kernel or $\tau_{\epsilon-5HT}$ (upper panel; M=1000). Latency time of rewarded trials at episode 20 (lower panel). Significance level (two-sample Welch's t-test) is measured against $\tau_{\epsilon-5HT}$ of 2 s (label above) and 5 s (label below). Shaded areas of the curves correspond to SEM.

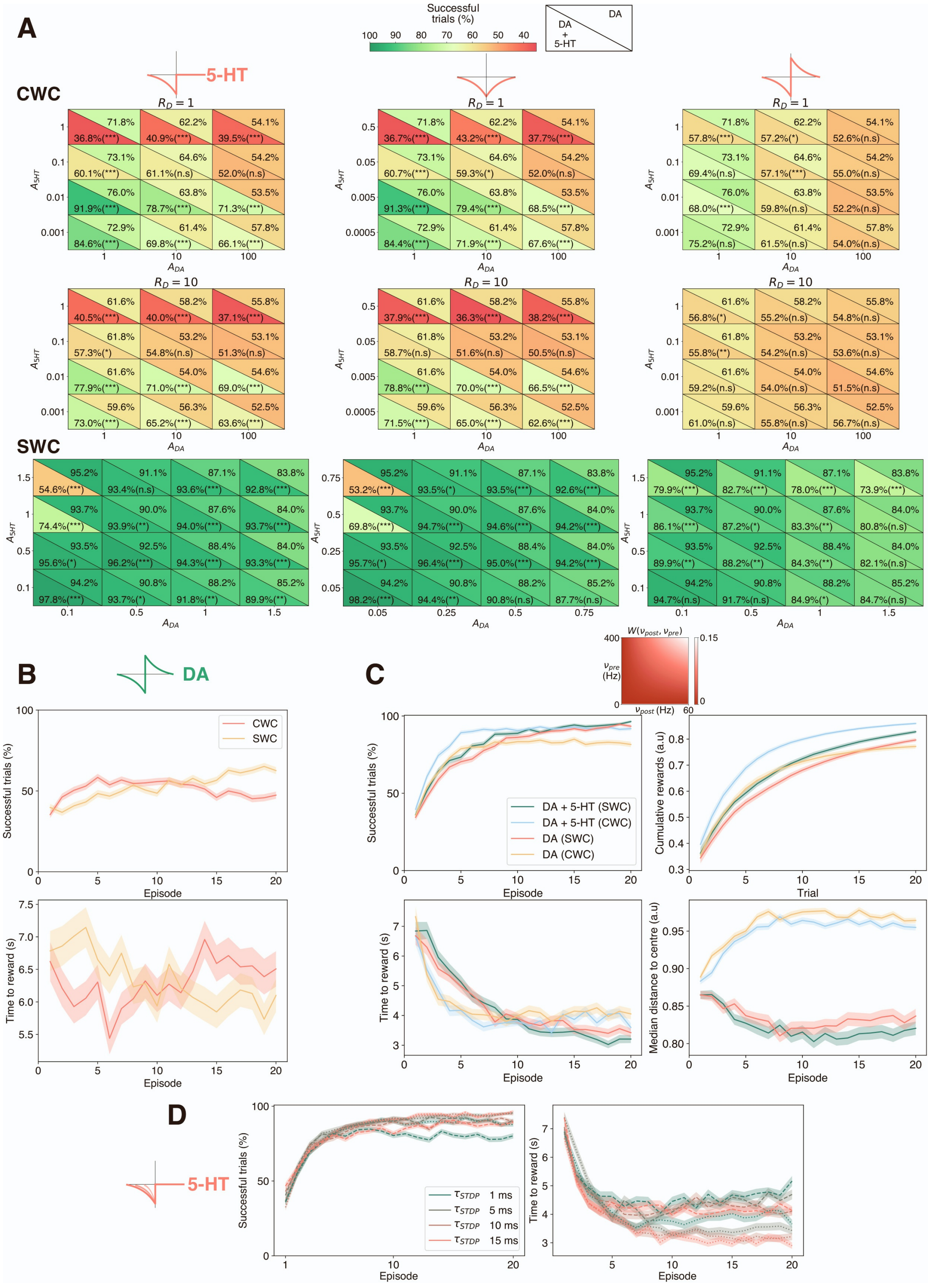

A
Successful trials (%)
100 90 80 70 60 50 40
DA
DA + 5-HT
5-HT
CWC
$R_D = 1$
$R_D = 10$
A_{5HT}
A_{DA}
SWC
B
DA
CWC
SWC
Successful trials (%)
Time to reward (s)
Episode
C
$W(\nu_{post}, \nu_{pre})$
ν_{pre} (Hz)
ν_{post} (Hz)
DA + 5-HT (SWC)
DA + 5-HT (CWC)
DA (SWC)
DA (CWC)
Successful trials (%)
Cumulative rewards (a.u)
Trial
Time to reward (s)
Median distance to centre (a.u)
Episode
D
5-HT
τ_{STDP} 1 ms
τ_{STDP} 5 ms
τ_{STDP} 10 ms
τ_{STDP} 15 ms
Successful trials (%)
Time to reward (s)
Episode

Figure S2: **Area of the STDP window modifies learning performance and a rate-based rule also models neuromodulated reinforcement. Related to Figure 2.**. **A** Heat map of the average percentage of successful trials at episode 20 for runs with DA and 5-HT (bottom left value) and DA-only (upper right value). The grid-search is done for different amplitudes of STDP windows being: exclusively anti-casual and negative (left), negative and symmetric with the same area (center), and the canonical STDP window (right). The latter impedes learning. For SWC the rate between reward amplitudes and other parameters were kept constant for CWC and SWC (see STAR Methods). Level of significance between DA and DA+5H-T is shown (M=1000 simulations, two-sample Student's t-test). **B** An symmetrical STDP window for DA hinders forward learning in the MWM task. Learning curve shown as the percentage of successful trials along episodes (above panel) and latency time to the platform of successful trials (lower panel) for M=500 simulations. **C** A rate-based rule reproduces the results of R-STDP in the MWM task. We employed a static BCM rule showing the same characteristics as the STDP window of DA and 5-HT (inset: window for an amplitude $A_{STDP} = 1$; see STAR Methods) to recreate the plots of ***Figure 1*** (M=500). **D**. STDP decay value of 5-HT does not affect learning performance. Percentage of successful trials along episodes (above panel) and latency times (lower panel). Neither SWC (dotted) nor CWC (dashed) significantly altered their performance for different decays of the exponential kernel of the STDP window (M=1000). Shaded areas correspond to SEM.

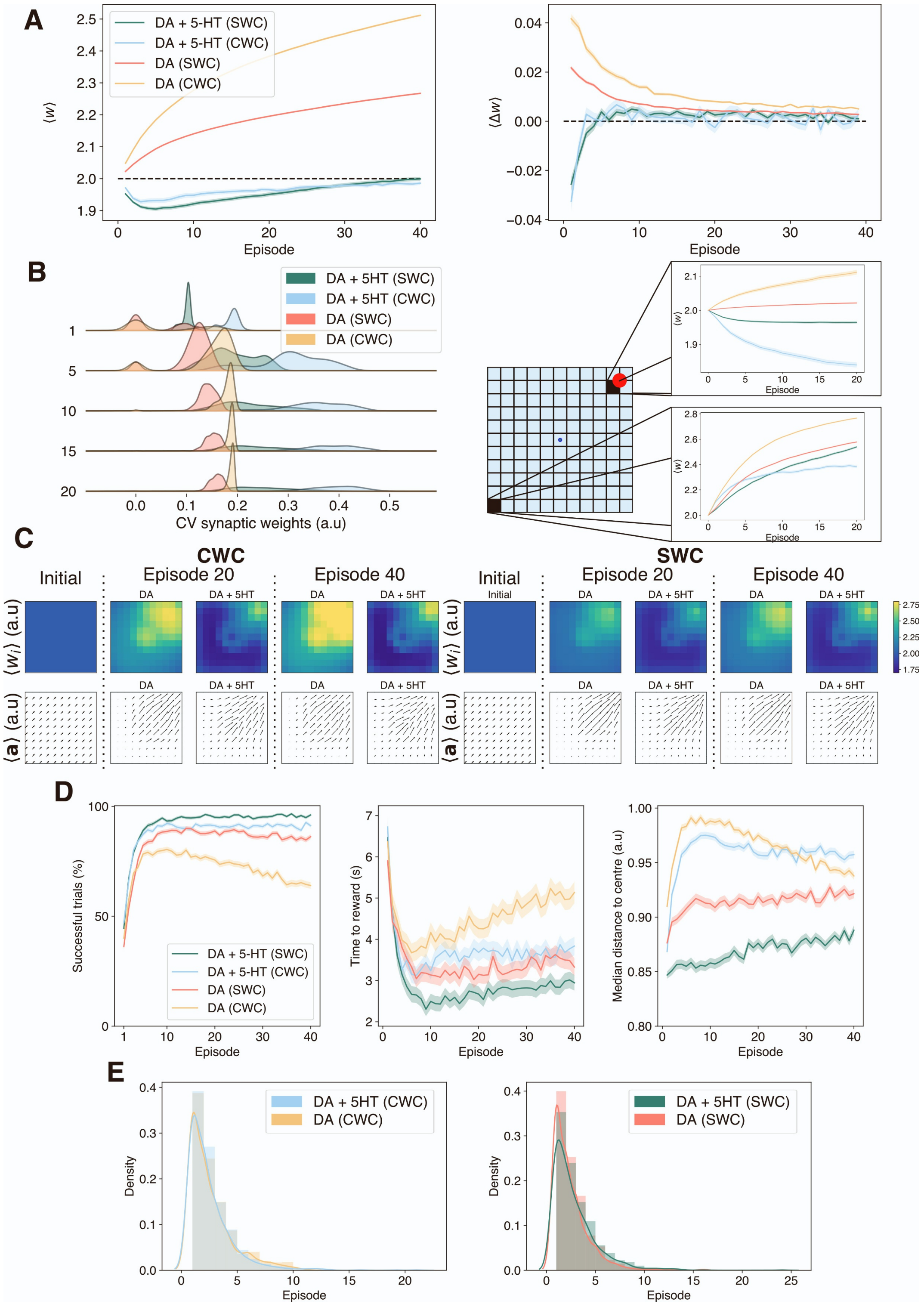

A
B
C
D
E
DA + 5-HT (SWC)
DA + 5-HT (CWC)
DA (SWC)
DA (CWC)
Episode
CV synaptic weights (a.u)
DA + 5HT (SWC)
DA + 5HT (CWC)
CWC
SWC
Initial
Episode 20
Episode 40
DA
DA + 5HT
⟨w_i⟩ (a.u)
⟨**a**⟩ (a.u)
Successful trials (%)
Time to reward (s)
Median distance to centre (a.u)
Density

Figure S3: **Synaptic weight changes and location dependencies and effects of SWC and CWC in the MWM task. Related to Figure 2.**. **A** Evolution and stability of synaptic weights in CWC and SWC. Average weight across place-action pairs (left) and mean weight change (right) along episodes. The dashed lines represents the initial weight and weight convergence (weight change $\Delta w = 0$), correspondingly. **B** Synaptic weight variability across the place field is more dispersed under 5-HT, and individual weights may fail to converge. Distribution of the CV of feed-forward synaptic weights (left panel) and mean weight evolution for two particular place cells (right panel), near the reward (upper inset) and opposite to it (lower inset), along episodes for each condition. CV corresponds to the ratio between the sample standard deviation of weights and its mean (see Quantification). The exemplary place cell plots show the initial position (blue dot) and platform position (red circle). Weights are bounded by clipping (see STAR Methods; M=1000). **C** Heat plots of the average feed-forward synaptic weights of place cells (above subplot) and action readouts for each episode (below) averaged along trials. Magnitudes of the policy plot are normalized by the maximal vector norm. For each condition and rule, the plots correspond to the initial time, episode 20 and 40 (M=1000). **D** Randomized initial positions do not affect spatial navigation performance in the MWM. Percentage successful trials (left panel), time-of-arrival (center) and median distance to the center (right). Initial positions were kept constant across episodes but were sampled from a uniform distribution in each trial (M=1000). The range of the SEM shows a higher inter-trial deviation than with a constant initial position in ***Figure 1***. **E** Addition of 5-HT does not alter exploratory behavior, depicted as first visit distribution. Comparison of the histograms of the first reward visit for 5-HT+DA and DA only in CWC ($JSD = 0.075$) and SWC ($JSD = 0.063$). Lines correspond to smoothing with kernel density estimation (M=1000). Shaded areas of plots correspond to SEM.

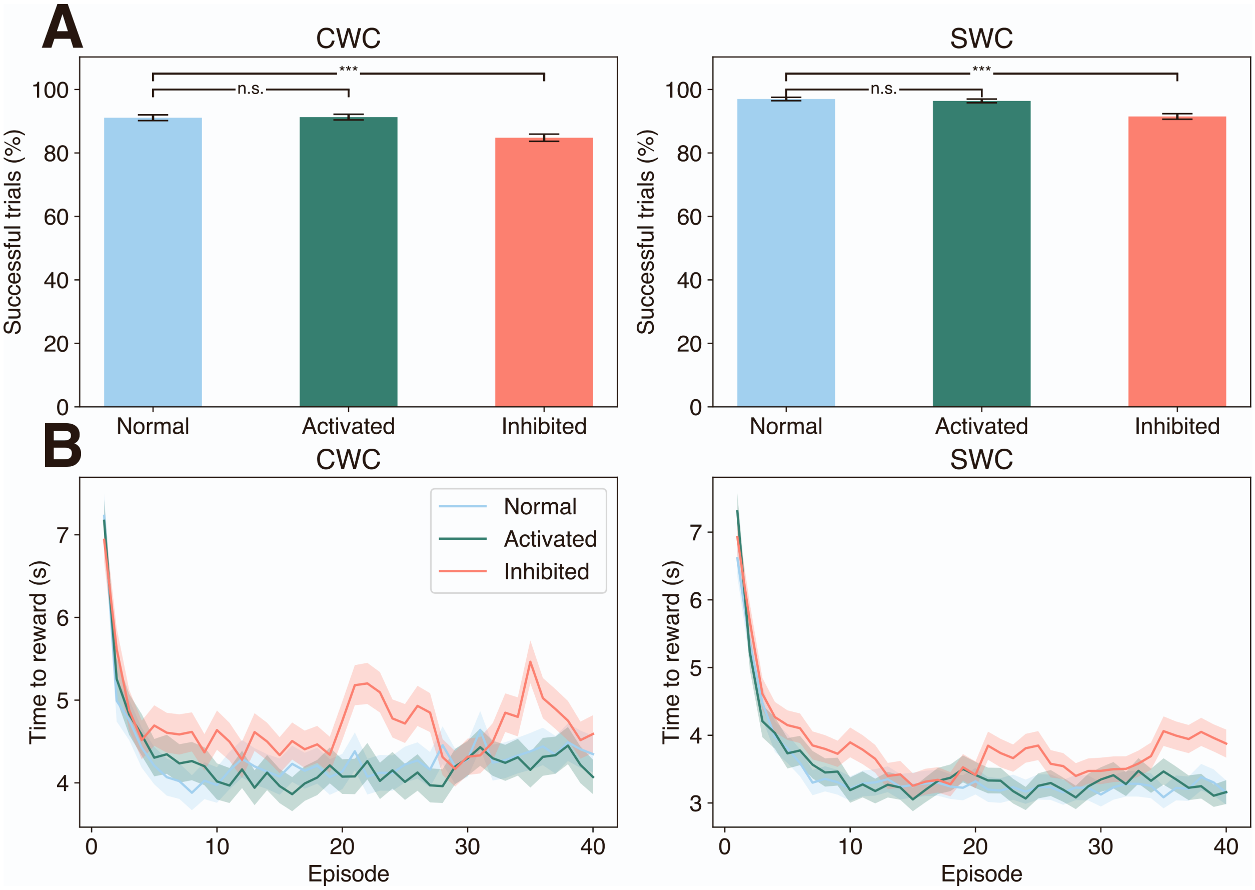

A
CWC
SWC
Successful trials (%)
n.s.
***
0
20
40
60
80
100
Normal
Activated
Inhibited
B
CWC
SWC
Time to reward (s)
Normal
Activated
Inhibited
Episode
0
10
20
30
40

Figure S4: **Optogenetic overactivation and inhibition of serotonergic activity modifies performance but does not influence latency. Related to Figure 3.**. **A** Bar plot of the percentage of successful trials at episode 40 for CWC and SWC. The error bars show the 95% confidence interval of the distribution. Statistical significance against the control is displayed above (two-sample Student's t-test).**B** Latency curves of successful agents under optogenetic modulation of 5-HT activation. SEM is plotted as regions around the mean (M=1000).
